# Protective effects of baicalin against L-glutamate-induced oxidative damage in HT-22 cells by inhibiting NLRP3 inflammasome activation via Nrf2/HO-1 signaling

**DOI:** 10.22038/IJBMS.2023.64318.14149

**Published:** 2023-03

**Authors:** Junyuan Li, Gang Wang, Yehao Zhang, Xiaodi Fan, Mingjiang Yao

**Affiliations:** 1 Institute of Basic Medical Sciences, Xiyuan Hospital of China Academy of Chinese Medical Sciences, No.1 Xiyuan Caochang, Haidian District, Beijing, 100091, China; 2 Key Laboratory of Pharmacology of Chinese Materia Medica, Beijing 100091, China; 3 Hubei Provincial Hospital of Integrated Chinese and Western Medicine, Wuhan, Hubei 430015, China; # These authors contributed eqully to this work

**Keywords:** Baicalin, L-glutamate, NLRP3 inflammasome, Nrf2/HO-1 signaling – pathway, Oxidative stress

## Abstract

**Objective(s):**

To explore the ability and underlying molecular mechanisms involved in the protective effects of Baicalin (BA) against L-Glutamate-induced mouse hippocampal neuron cell line HT-22.

**Materials and Methods:**

The cell injury model of HT-22 cells was induced by L-glutamate, and cell viability and damage were detected by CCK-8 and LDH assays. Generation of intracellular reactive oxygen species (ROS) was measured by DCFH-DA *in situ* fluorescence method. The SOD activity and MDA concentration in the supernatants were determined by WST-8 and colorimetric method, respectively. Furthermore, Western blot and real-time qPCR analysis were utilized to detect the expression levels of the Nrf2/HO-1 signaling pathway and NLRP3 inflammasome proteins and genes.

**Results:**

L-Glutamate exposure induced cell injuries in HT-22 cells, and the concentration of 5 mM L-Glutamate was chosen to be the modeling condition. Co-treatment with BA significantly promoted cell viability and reduced LDH release in a dose-dependent manner. In addition, BA attenuated the L-Glutamate-induced injuries by decreasing the ROS production and MDA concentration, while increasing the SOD activity. Moreover, we also found that BA treatment up-regulated the gene and protein expression of Nrf2 and HO-1, and then inhibited the expression of NLRP3.

**Conclusion:**

Our study found that BA could relieve oxidative stress damage of HT-22 cells induced by L-Glutamate, and the mechanism might be related to the activation of Nrf2/HO-1 and inhibition of NLRP3 inflammasome.

## Introduction

Glutamate, the most abundant endogenous excitatory neurotransmitter in the brain and spinal cord of vertebrates, is thought to be mediated primarily by activating three main types of ionotropic receptors and several types of metabolic receptors associated with G-proteins, and plays an indispensable role in the differentiation, survival, and migration of neurons (1). However, pathologic states caused by cerebrovascular and neurodegenerative diseases may result in a sharp rise in the concentration of glutamate in the extracellular fluid, and thus exerts heavy excitotoxicity, which results in the injury and death of neurons (2, 3). Glutamate cytotoxicity in neurons is associated with reactive oxygen species (ROS) generation via receptor-mediated excitotoxicity or non-receptor-mediated oxidative toxicity. In neurons, the overt activation of ionotropic glutamate receptors leads to an elevation in Ca^2+^ fluxes and catabolic enzyme activities, the increase of ROS production which ultimately results in neurodegeneration (4, 5). Another pathway through which glutamate induces cytotoxicity is called glutamate oxidative toxicity. Indeed, a high level of extracellular glutamate inhibits the cellular uptake of cystine via the cystine/glutamate transport system, which subsequently leads to the depletion of intracellular glutathione (GSH) and a detrimental accumulation of excess ROS, thus stimulating the expression of inflammatory factors, culminating in oxidative stress and cell death (6, 7). 

Oxidative stress is actually characterized by increased ROS production (8). The oxidative neuronal cytotoxicity is thought to be mediated by Nuclear factor E2-related factor 2 (Nrf2), a key transcription factor that regulates the expression of anti-oxidant proteins such as heme oxygenase-1 (HO-1), superoxide dismutase (SOD), and GSH (9), and is activated in response to ROS to protect cells against oxidative stress. HO-1 is a critical stress protein involved in the defense mechanism against oxidative damage and inflammatory injury (10), and its activation is a common feature of neurodegenerative diseases. 

Inflammasome is composed of pattern recognition receptor (PRR), caspase-1, and apoptosis-associated speck-like protein (ASC), which has been observed in monocytes and macrophages. Inflammasome maintains the balance of pro-inflammatory and anti-inflammatory factors, and their activation is associated with the pathogenesis of certain inflammatory disorders such as Alzheimer’s disease (11). The nod-like receptor pyrin-containing pyrin domain 3 (NLRP3) inflammasome is the most widely studied inflammasome, which is essential to protect against exogenous infections.

Baicalin (BA) is a natural flavonoid extracted from* Scutellaria baicalensis*, a widely used Chinese herbal medicine with strong neuroprotective abilities in central nervous system diseases (12-14). It has various pharmacological effects including anti-oxidant, anti-inflammatory, and antiapoptotic (15-17), and has been proven experimentally and clinically for treating many cerebrovascular and neurodegenerative diseases. HT-22 cells, an immortalized progenitor neuronal cell line derived from the mouse hippocampus that lack functional ionotropic glutamate receptors, have been widely used as an *in vitro* model in studying the mechanisms of oxidative stress-induced neuronal death (18, 19).

In this study, we found that BA could inhibit L-glutamate-induced oxidative damage in HT-22 cells by inhibiting NLRP3 inflammasome activation via Nrf2/HO-1 signaling, and our results suggest that BA may regulate inflammation and oxidative stress in neuronal injury, and therefore has potential clinical transformation relevance. 

## Materials and Methods

***Reagents***

Dulbecco’s modified Eagle’s medium (DMEM), Fetal bovine serum (FBS), and trypsin-EDTA were purchased from Gibco (Life Technologies, Grand Island, NY, USA). Penicillin and streptomycin were purchased from Hyclone (Logan, UT, USA). PBS and L-glutamate (L-Glu) were purchased from Solarbio (Beijing, China). DMSO was purchased from Aladdin (Shanghai, China). N-acetyl-L-cysteine (NAC) was purchased from Sigma Chemical Co (St. Louis, MO, USA), and BA was purchased from MedChemExpress (NJ, USA).

***Cell culture and treatments***

The HT-22 cell line was purchased from Procell Life Science&Technology (Wuhan, Hubei, China) and cultured with DMEM containing 10% FBS, 1% penicillin/streptomycin in a humidified atmosphere with 5% CO_2_ at 37 °C. Cells were subcultured once every 2~3 days and prepared for experiments when in log-phase growth. The stock solution of L-glutamate was prepared at the concentration of 30 mM in DMEM and filtered through a 0.2 μm filter.

Cells were seeded in 96-well plates at a density of 5000 cells per well and were cultured for 24 hr. The medium in each well was discarded and replaced with various concentrations of L-glutamate (1, 2, 4, 5, 6, 8, 10, 12, and 15 mM) that were diluted in the culture medium, and then cultured for another 24 hr. Experiments of the control group were carried out by adding a culture medium without L-glutamate. Besides, different concentrations of BA (1, 2, 4, 8, and 16 μM) were simultaneously added to the culture medium with L-Glutamate for co-culturing. 

***CCK-8 assay***

Cell viability was detected using a CCK-8 Assay Kit (Dojindo, Kumamoto, Japan). Twenty-four hours after various treatments, the supernatants in each well were discarded and the cells were washed twice with PBS. Then, a reaction mixture with 100 μl medium and 10 μl CCK-8 solution was added to each well and was incubated for an additional 2 hr at 37 °C. Absorbance was measured at 450 nm wavelength on a microplate reader. Each group was prepared in 6 multiple wells and 3 independent experiments. The relative cell viability was expressed as a percentage of the control cells that were not treated with L-glutamate.

***LDH assay***

The release of LDH indicates the integrity of the cell membrane (20). LDH activity was measured with an LDH assay kit (Beyotime, Shanghai, China) according to the manufacturer’s instructions. Cells were seeded in 96-well plates at a density of 5000 cells per well and cultured at 37 °C for 24 hr. After co-culture with BA of different concentrations (4 μM, 8 μM, and 16 μM), the cell supernatant of each well was collected and reacted with substrate solution at 37 °C for 30 min. The absorbance was measured at 450 nm wavelength on a microplate reader. Each group was prepared in 6 multiple wells and 3 independent experiments. The results were expressed as relative to the control group.

***SOD activity and MDA concentration***

To determine the degree of oxidative stress, the activity of SOD and the concentration of MDA were measured. SOD activity was tested using a SOD kit (Beyotime, Shanghai, China) by WST-8 assay according to instructions. The content of MDA was tested using an MDA kit (Beyotime, Shanghai, China) by the colorimetric method according to the instructions.

***ROS production***

ROS production was detected by DCFH-DA *in situ* fluorescence assay (Beyotime, Shanghai, China). Cells were seeded in a confocal dish at a density of 0.5×10^5 ^ml^-1^ when in log-phase growth and cultured for 24 hr at 37 °C. After being co-cultured with different concentrations of BA for another 24 hr, cells were washed with PBS and incubated with 10 μM DCFH-DA for 30 min. Then the fluorochrome was discarded and washed with PBS. The production of ROS in each group was observed under a confocal microscope (LSM 510 META, ZEISS, Germany) with ex: 488 nm/em: 525 nm.

***Western blotting***

The total protein of HT-22 cells was extracted using high-efficiency RIPA lysate (Solarbio, Beijing, China). Protein samples of all groups were tested by Quick Start Bradford assays (Bio-Rad, USA). The proteins were loaded on 10% sodium dodecyl sulfate-polyacrylamide gel electrophoresis (SDS-PAGE) gels and then transferred to the PVDF membrane. The membrane was blocked with 5% skimmed milk for non-specific protein binding for 2 hr at room temperature. The membrane was washed with Tris-Buffered Saline with 1% Tween 20 (TBST, ZSGB-BIO, Beijing, China) three times. Next, it was incubated with the following primary antibodies overnight at 4 °C: NLRP3 (1:1000, Proteintech, Wuhan, China), Nrf2 (1:1500, Proteintech, Wuhan, China), HO-1 (1:1500, Proteintech, Wuhan, China), IL-1β (1:1000, Proteintech, Wuhan, China), TXNIP (1:500, WanleiBio, Shenyang, China), β-actin (1:3000, Cell Signaling Technology, MA, USA), and GAPDH (1:3000, Proteintech, Wuhan, China). Lastly, it was incubated with the secondary antibodies for 1 hr (1:20000, Huaxingbio, Beijing, China) at room temperature. The blotted protein bands were reacted using an enhanced chemiluminescence (ECL) reagent (Beyotime, Shanghai, China) and captured by ChemiDoc XRS+ System (BIO-RAD, USA). The visualized protein bands were further quantified with ImageJ software (Version 1.52a).

***Real-time qPCR***

The total RNA was extracted from the HT-22 cells using TRIzol reagent (Solarbio, Beijing, China). The mRNA concentrations were measured using a SMA1000 UV Spectrophotometer (Merinton, Beijing, China). Total RNA was reverse transcribed into cDNA by using Primescript Rt Master Mix according to the manufacturer’s protocol (TOYOBO, Tokyo, Japan). SYBR® Green Real-time PCR Master Mix (Sigma, St. Louis, MO, USA) was used to determine the mRNA level of the gene of interest by employing StepOnePlus Real-Time PCR System (ABI, CA, USA). The relative mRNA levels were normalized to the housekeeping gene β-actin. Primers used in the study were: β-actin (forward 5’-CTACCTCATGAAGATCCTGACC-3’ and reverse 5’- CACAGCTTCTCTTTGATGTCAC-3’), Nrf2 (forward 5’- CAGCCATGACTGATTTAAGCAG-3’ and reverse 5’- CAGCTGCTTGTTTTCGGTATTA-3’), and NLRP3 (forward 5’- GAGCTGGACCTCAGTGACAATGC-3’ and reverse 5’- ACCAATGCGAGATCCTGACAACAC-3’). Relative mRNA levels of indicated genes were analyzed using the 2^-ΔΔCt ^method. 

***Statistical analysis***

Each experiment above was repeated three times, and finally, the average value was taken for statistical analysis. SPSS 19.0 was used for statistical analysis and Graphpad prism 5.0 was used for diagramming. The data were expressed as Mean±SEM, and the comparison between groups was performed by one-way ANOVA and Student’s t-test. *P*<0.05 was considered to be statistically significant.

## Results

***Effects of L-glutamate on normal HT-22 cells***

**Figure 1:**
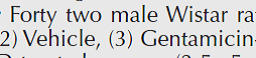
HT-22 cells were treated with varying concentrations of L-Glutamate for 24 hr, and cell viability was detected by CCK-8 assay. The data are expressed as mean±SEM (n = 6). #*P*<0.05 and ##*P*<0.01 versus control group, *P<0.05 and **P<0.01 versus model group

HT-22 cells were exposed to a series of concentrations of L-Glutamate and co-cultured for 24 hr. We found out that L-glutamate reduced cell viability in a dose-dependent manner (Figure 1) with an IC_50_ of 5.33 mM. Therefore, 5 mM L-glutamate was chosen as the modeling condition in the following experiment.

***Effects of BA on normal HT-22 cells***

**Figure 2:**
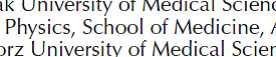
HT-22 cells were treated with varying concentrations of Baicalin (BA), and cell viability was detected by CCK-8 assay. The data are expressed as mean±SEM (n = 6). #*P*<0.05 and ##*P*<0.01 versus control group, *P<0.05 and **P<0.01 versus model group

HT-22 cells were incubated with different concentrations of BA for 24 hr. As illustrated in Figure 2, the viability of the HT-22 cells did not diminish upon treatment with 1 to 16 μM BAconcentrations (*P*>0.05). It could be determined that BA at the experimental concentrations causes no toxic effect on HT-22 cells. 

***Effects of BA on L-glutamate-induced HT-22 cell viability***

**Figure 3:**
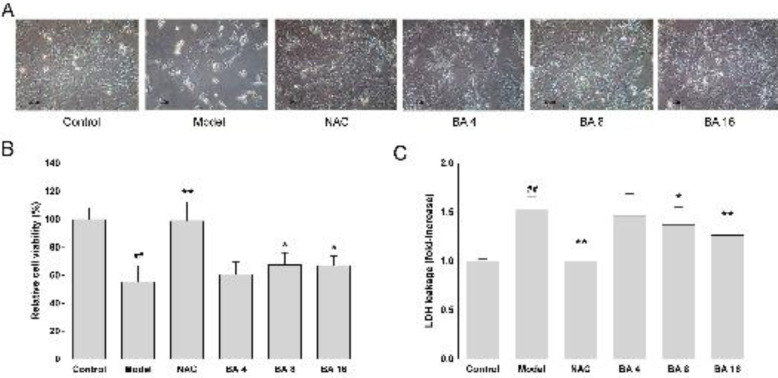
HT-22 cells were treated with 5 mM glutamate and BA (4 μM, 8 μM, and 16 μM) for 24 hr. (A) Morphology of treated cells was observed under a microscope (scale bar is 20 μm). (B) Cell viability was detected using the CCK-8 assay. (C) Cell death was measured using LDH assay. The data are expressed as mean±SEM (n = 6). #*P*<0.05 and ##*P*<0.01 versus non-treated control group, *P<0.05 and **P<0.01 versus the model group

BA: Baicalin; LDL: Lactate dehydrogenase

HT-22 cells were co-cultured with 5 mM L-glutamate and BA (4 μM, 8 μM, and 16 μM) for 24 hr, and the control group was carried out by culturing with a normal medium. To determine the viability of HT-22 cells, we recorded the morphological features of the HT-22 cells induced by L-glutamate after subsequent treatment by BA. As shown in Figure 3A, compared with the control group, cells of the model group showed shrinkage, roundness, and broadened intercellular gaps, and BA effectively alleviated the above situation.

The CCK-8 assay further indicated that cell activity was significantly decreased in the model group (*P*<0.01, Figure 3B). Compared with the model group, cell viability significantly increased after being treated with middle and high dose BA (*P*<0.05, Figure 3B). Meanwhile, the LDH release quantity in the model group significantly increased compared with the control group (*P*<0.05, Figure 3C). Compared with the model group, the LDH release quantity significantly decreased after treatment with middle and high dose BA (*P*<0.05, Figure 3C; *P*<0.01, Figure 3C). These results indicated that BA can alleviate L-glutamate-induced HT-22 cell injury.

***Effects of BA on L-glutamate-induced oxidative stress in HT-22 cells***

**Figure 4:**
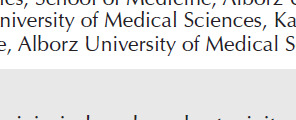
HT-22 cells were treated with 5 mM glutamate and BA (4 μM, 8 μM, and 16 μM) for 24 hr. (A) Fluorescent images were acquired using a fluorescent microscope (scale bar is 20 µm). (B) Quantitative analysis of the level of ROS. Data were expressed as a relative ROS level of the control group. (C) Quantitative analysis of the level of MDA. (D) Quantitative analysis of the level of SOD. The data are expressed as mean±SEM (n = 6). #*P*<0.05 and ##*P*<0.01 versus control group, *P<0.05 and **P<0.01 versus model group

BA: Baicalin; SOD: Superoxide dismutase; ROS: Reactive oxygen species; MDA: Malondialdehyde

SOD is one of the major anti-oxidant enzymes that catalytically converts the superoxide radical to hydrogen peroxide (H_2_O_2_) (21), and MDA is widely accepted as a biomarker of oxidative stress, namely lipid peroxidation (22). Oxidative stress was induced by excessive ROS, and we measured cellular ROS with a DCFH-DA assay kit. The trends of these indexes tend to be dose-dependent. As shown in Figure 4, the level of ROS significantly increased in the model group compared with those in the control group (*P*<0.01, Figure 4A, B). However, the level of ROS decreased after being treated with all three doses of BA (*P*<0.05, Figure 4A, B). Meanwhile, the expression of MDA was consistent with ROS. The MDA level significantly increased after treatment with L-Glutamate (*P*<0.05, Figure 4 C), and all three doses of BA have reversed the trend (*P*<0.01, Figure 4C). Reversely, the level of SOD decreased in the model group compared with those in the control group (*P*<0.01, Figure 4D) and increased after being treated with a high dose of BA (*P*<0.05, Figure 4D). These results indicate that BA significantly reduced oxidative damage in HT-22 cells caused by L-glutamate.

***Effects of BA on expression of Nrf2/HO-1 signaling pathway and NLRP3 inflammasome in L-Glutamate-induced HT-22 cells***

**Figure 5:**
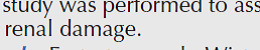
HT-22 cells were treated with 5 mM glutamate and Baicalin (BA) (4 μM, 8 μM, and 16 μM) for 24 hr. (A) Protein levels of Nrf2, HO-1, and β-actin were measured by western blot analysis. (B) Protein levels of TXNIP, NLRP3, IL-1β, and GAPDH were measured by western blot analysis. (C–D) Quantitative results of protein levels of Nrf2 and HO-1. (E–G) Quantitative results of protein levels of TXNIP, NLRP3, and IL-1β. The data are expressed as mean±SEM (n = 3). #*P*<0.05 and ##*P*<0.01 versus sham group, *P<0.05 and **P<0.01 versus model group

Nrf2: Factor E2-related factor 2; HO-1: Hemeoxygenase-1

**Figure 6:**
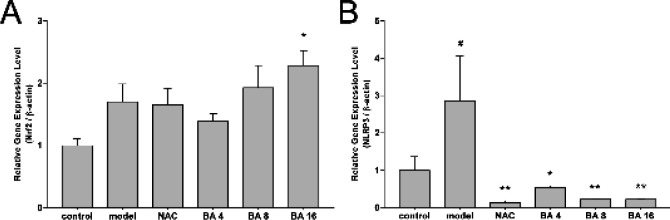
HT-22 cells were treated with 5 mM glutamate and Baicalin (BA) (4 μM, 8 μM, and 16 μM) for 24 hr. The expression of mRNA of Nrf2 and NLRP3 in HT-22 cells was examined by Real-time qPCR. The data are expressed as mean±SEM (n= 3) #*P*<0.05 and ##*P*<0.01 versus the non-treated control group, *P<0.05 and **P<0.01 versus the model group

To investigate the mechanism of BA in HT-22 cells after being treated with glutamate, the effects of BA on the Nrf2/HO-1 signaling pathway and NLRP3 inflammasome were measured. As is shown in Figure 5, after being treated with glutamate, the expression of Nrf2, HO-1 was increased in the model group, and continued to increase in the high-dose BA group (*P*<0.05, Figure 5A, C, D). However, there is no statistical significance between the sham group and the model group of Nrf2. Meanwhile, the expression of NLRP3, TXNIP, and IL-1β was increased in the model group, and high-dose BA treatment reversed the trend (*P*<0.05, Figure 5 E, F, G). However, there is no statistical significance between the sham group and the model group of the IL-1β. We also examined the effects of BA intervention on the mRNA expression of Nrf2 and NLRP3 (Figure 6A, B), and the results were consistent with those of western blotting. The expression of Nrf2 was increased in the model group, and continued to increase in the high-dose BA group (*P*<0.05, Figure 6A). The expression of NLRP3 was increased in the model group (*P*<0.05, Figure 6B), and all three doses of BA reversed the trend (*P*<0.05, Figure 6B). These results indicate that BA can alleviate glutamate-induced oxidant damage to HT-22 cells via the Nrf2/HO-1 signaling pathway and NLRP3 inflammasome. 

Nrf2: Factor E2-related factor 2

## Discussion

Oxidative damage was thought to be a common early event in neurodegenerative diseases. The production of ROS is caused by the imbalance of the production and clearance of oxygen-free radicals in the body or cells. HT-22 cells are highly sensitive to glutamate, which could produce a large amount of ROS and lipid peroxidation products such as MDA, as well as inflammatory immune cytokines and regulators such as IL-6, TNF-α, IL-1β, and NO due to over-excitation of glutamate receptors. Thus, HT-22 cells are frequently used as a neuronal model system for studying glutamate-induced toxicity and identifying molecular mechanisms associated with anti-oxidative or anti-inflammatory chemical compounds (23). In the present study, we demonstrated the effect of BA on the Nrf2/HO-1 signaling pathway and NLRP3 inflammasome in oxidative stress injury. In L-glutamate-induced oxidative damage HT-22 cell model, BA treatment showed a significant inhibitory effect on the activation of ROS and MDA and promoted the production of SOD, which may be mediated by Nrf2/HO-1 signaling pathway and NLRP3 inflammasome.

Long-term activation of glutamate receptors will lead to a series of neurotoxicity, ultimately leading to neuronal function loss and cell death. Herein, the effects of different doses of glutamate in HT-22 cells were assessed. And the IC_50_ of HT-22 cells exposure to L-glutamate was about 5 mM, which was consistent with previous studies (24). Meanwhile, the toxicity of BA to HT-22 cells was detected according to previous studies (25-27). In the current study, 16 μM BA was the most effective dose against oxidative stress. Actually, BA has shown anti-oxidant stress activity in *in vivo *and* in vitro* models (28-31). Glutamate treatment elevated MDA and ROS levels and reduced SOD levels in HT-22 cells, indicating that oxidative stress occurs in HT-22 cells. The levels of MDA and SOD can reflect the damage degree of lipid peroxidation and the ability to scavenge oxygen free radicals. The former is the final product of lipid peroxidation and indirectly reflects the degree of oxidative damage to the body, while the latter is the key enzyme that plays an anti-oxidant role in the scavenging system of the body and protects cells from oxidative damage (32). Our results showed that BA protected against glutamate-induced cell death by enhancing anti-oxidant systems.

Studies revealed that activation of nuclear transcription factor E2-related factor 2 (Nrf2) and heme oxygenase 1 (HO-1) can alleviate oxidative stress damage (33, 34). Nrf2 is a key coordinator for improving the intracellular responses to several oxidative and inflammatory insults. And the Nrf2 activators have become a potential therapeutic strategy for neurodegenerative diseases (35) Under stress conditions, Nrf2 is released from Keap1 repression, translocates to the nucleus, where it interacts with anti-oxidant response elements (ARE) to drive the endogenous anti-oxidant system, and promotes the expression of cytoprotective genes to restore the redox balance in cells and resist oxidative stress damage (36, 37). Oxidative stress is one of the main causes of glutamate-mediated neuronal damage, while in glutamate stimulation, the Nrf2/HO-1 signaling pathway can be activated to start the endogenous protection mechanism (38). Activation of the Nrf2/HO-1 signal can reduce the production of MDA and ROS in hypoxic-ischemic brain injury rats (39). Nrf2 knockout mice showed significantly reduced anti-oxidant stress activity and HO-1 expression (40). The Nrf2/HO-1 signaling pathway has been shown to be an endogenous anti-oxidant (41). It has shown good therapeutic effects in various animals and *in vitro* models of inflammation and oxidative stress (42-44). BA effectively alleviates oxidative stress and apoptosis by activating Nrf2/HO-1 signaling pathway and protects the thymus from *Mycoplasma gallisepticum* infection (45). In the LPS-induced severe lung injury animal model, BA has shown a protective effect on lung injury by activating the Nrf2/HO-1 signaling pathway (46). In this study, glutamate stimulation significantly increased the protein and mRNA expression of Nrf2 and HO-1 in HT-22 cells. However, BA treatment has reversed the trend, which elucidated its neuroprotective activity.

Furthermore, several studies have shown that Nrf2/HO-1 signaling-associated anti-oxidant capacity could then inhibit NLRP3 inflammasome-mediated inflammatory injury (47, 48). NLRP3 inflammasome is a polymeric protein complex that triggers a series of related inflammatory responses in the body that can be activated by reactive oxygen species (ROS) accumulation and participate in mediating the secretion of proinflammatory cytokines interleukin-1β (IL-1β) and IL-18 (49). ROS accumulation intensified the activation of NLRP3 inflammasome (50-53). ROS is an upstream signal of NLRP3 inflammasome activation, and NLRP3 ligand TXNIP is highly sensitive to ROS (54). Research showed (55) that ROS and MDA levels as well as TXNIP, NLRP3, and IL-1β levels were increased, and the TXNIP/NLRP3 inflammasome pathway was activated in high glucose-treated rat mesangial cells. TXNIP knockout inhibits the TXNIP/NLRP3 inflammasome pathway activity induced by high glucose and reduces oxidative stress injury. Glutamate stimulation induces hyperactivation of ROS and TXNIP/NLRP3 inflammasome (56). Our data showed that BA treatment down-regulated the expression of NLRP3, TXNIP, and IL-1β in glutamate-induced HT-22 cells. Since BA showed an inhibitory effect in the activation of NLRP3 inflammasome in an animal model (57), it is promising that BA exerts protective effects against inflammatory injury through NLRP3 inflammasome.

BA plays a direct or indirect therapeutic role in the treatment of brain injury (58), nerve cell injury (59), tumors (60), and other diseases through anti-inflammatory and anti-oxidative stress. Flavonoids relieve neurodegenerative disease injury by suppressing the expression of pro-inflammatory mediators and inhibiting neuroinflammation by reducing cytokine release (61-63). Anti-oxidant is one of the main functions of BA, and the core mechanism lies in activating Nrf2 factors, thus affecting the transcription of anti-oxidant protein reaction elements. In this study, an *in vitro* model was established by co-culturing L-Glutamate with HT-22 cells, and significant oxidative stress damage on the cells was observed. The results showed that BA treatment inhibited the activation of the NLRP3 inflammasome, meanwhile, the Nrf2/HO-1 signaling pathway was activated.

## Conclusion

In this study, the anti-oxidant activity of BA was observed in the L-Glutamate-induced HT-22 cells, and the results showed that BA enhanced the activity of SOD, inhibited the levels of oxidative mediators (ROS and MDA) and inflammatory genes (NLRP3) in HT-22 cells, further confirming that BA intervenes endogenous anti-oxidant activity and reverses the oxidative damage via the Nrf2/HO-1 signaling pathway and NLRP3 inflammasome. However, whether BA directly regulates Nrf2 factors or indirectly through other pathways remains to be further explored.

## Authors’ Contributions

JYL, GW, MJY designed the study; JYL, GW, MJY performed the experiments; JYL, GW, MJY, XDF and YHZ contributed to manuscript preparation. All authors have read and approved the final manuscript.

## Conflicts of Interest

The authors declare that no conflict of interest exists.
